# General anesthesia enhances lesion quality and ablation efficiency of circumferential pulmonary vein isolation

**DOI:** 10.1002/joa3.12960

**Published:** 2023-11-27

**Authors:** Kexin Wang, Caiyi Jin, Hongwu Chen, Gang Yang, Hailei Liu, Zidun Wang, Xiaohong Jiang, Weizhu Ju, Minglong Chen

**Affiliations:** ^1^ Division of Cardiology The First Affiliated Hospital of Nanjing Medical University Nanjing China

**Keywords:** atrial fibrillation, catheter ablation, conscious sedation, general anesthesia, pulmonary vein isolation

## Abstract

**Background:**

Pulmonary vein isolation (PVI) is the cornerstone of atrial fibrillation (AF) ablation. General anesthesia (GA) resolves the problem of pain intolerability and provides regular respiratory mode which might improve the catheter maneuverability of AF ablation. This study aims to compare the procedural performance of PVI under GA versus conscious sedation (CS) from multiple perspectives.

**Methods:**

A total of 36 consecutive patients undergoing first AF ablation under GA were enrolled in GA group. Another 109 patients receiving AF ablation under CS in the same period were selected as the control group. After propensity score matching, 29 matched pairs with similar baseline characteristics were available for further analysis. The AIFV (using AI to analyze the raw data from CARTO3 system) system was used to evaluate six procedural parameters in each PVI procedure.

**Results:**

Compared with CS, PVI under GA had a significantly shorter total PVI time (51.4 min vs. 67.8 min; *p* = .003) and higher radiofrequency ratio (62.6% vs. 55.8%; *p* = .032). The number of gaps (1.0 vs. 3.0; *p* < .001) and the rate of break point were significantly lower in the GA group. GA was also associated with a higher effective ablation‐index ratio (87.5% vs. 74.1%; *p* < .001) and effective force‐over‐time ratio (85.3% vs. 69.2%; *p* = .001). After a medium follow‐up time of 24 months, 12/29 (41.4%) patients in the CS group and 6/29 (20.7%) patients in the GA group suffered from AF recurrence (*p* = .156).

**Conclusions:**

GA improves the lesion quality and procedural efficiency of PVI from multiple perspectives evaluated by the AIFV system.

## Graphical abstract

A high‐quality pulmonary vein isolation is the foundation for improving the single‐procedure success rate of AF ablation. AIFV is a novel system designed to describe the overall quality for the two pulmonary vein circles. General anesthesia can safely improve the PVI lesion quality and procedural efficiency from multiple perspectives evaluated by AIFV system.

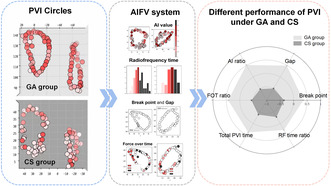

## 1 INTRODUCTION

Pulmonary vein isolation (PVI) is the cornerstone of atrial fibrillation (AF) ablation.^1^ It is widely recognized that optimizing lesion quality and ensuring contiguous, transmural, and sustainable PVI lesions are crucial for achieving a higher success rate in a single procedure.^2^, ^3^ Much effort and tremendous progress have been made to achieve the stable contact of catheter and transmural lesion, such as intubation and high‐frequency ventilation,^4^ deflectable long sheath,^5^ and force sensing catheter,^6^ et al. General anesthesia (GA) can eliminate patient movement, regulate respiratory rate and depth, thus may theoretically allow better catheter stability and more adequate contact force, which may potentially have impact on the effectiveness of AF ablation.^7^, ^8^, ^9^ To date, there is a lack of objective data regarding comprehensive evaluation of the performance of PVI under GA compared with that under the conscious sedation (CS). In this study, we employed a novel AIFV system that included six main criteria to comprehensively evaluate the quality of PVI and the efficiency of ablation under GA compared with CS from multiple perspectives.

## 2 METHODS

### 2.1 Study population

Consecutive symptomatic AF patients underwent their first AF ablation under general anesthesia in the First Affiliated Hospital with Nanjing Medical University between June 2020 and June 2021 were enrolled in the study. The control group comprised patients who received AF ablation by the same group of electrophysiologists during the same period under conscious sedation. The exclusion criteria were as following: (1) age < 18 or > 80 years; (2) patients with previous ablation history; and (3) unwillingness to follow‐up.

The study was approved by the Institutional Ethical Review board. All patients provided written informed consent.

### 2.2 Anesthesia protocols

In GA group, induction of anesthesia was achieved by propofol 2 mg/kg, sufentanil 0.2 ~ 0.5 ug/kg, and rocuronium 0.6 mg/kg. The airway was secured by endotracheal intubation. Anesthesia was maintained with propofol 0.03 ~ 0.05 mg/kg/min combined with sevoflurane 1 ~ 2%. The depth of anesthesia was adjusted to a range of bispectral index (BIS) between 40 and 60. The patients were ventilated with a tidal volume (Vt) of 7 ~ 8 mL/kg and a respiratory rate of 10 ~ 12 breaths/min. All these procedures were performed by an experienced anesthesiologist.

In CS group, ablation was performed under CS (with dexmedetomidine and fentanyl) and local anesthesia (with lidocaine).

### 2.3 Catheter ablation of atrial fibrillation

All patients were administrated novel oral anticoagulants for at least 3 weeks preablation. Left atrial thrombus was routinely excluded by transesophageal echocardiography before ablation. All anti‐arrhythmic drugs were ceased for at least five half‐lives before the procedure.

During the procedure, two 8.5‐F 65 cm long sheaths (SL1, St. Jude Medical, Inc.) were advanced to the left atrium through standard trans‐septal puncture. After the trans‐septal punctures, intravenous heparin was administered to maintain an activated clotting time of 300–350 s. A three‐dimensional anatomical shell of the left atrium was constructed using a mapping catheter (Pentaray, Biosense Webster, Inc.) guided by a CARTO3 system (Biosense‐Webster Inc.). All the procedures were performed by a team of six well‐experienced physicians, all with over 300 personal AF ablation procedures completed and over 20 instances of SmartTouch catheter use.

AF ablations were performed by a standardized approach.^10^, ^11^ An ablation index (AI)‐guided circumferential pulmonary vein isolation (CPVI) was routinely performed (power 30–45 W, contact force 5–30 g) using a SmartTouch catheter (Biosense Webster, Inc.). Point‐by‐point RF delivery was performed to produce a contiguous circle with a tag size of 4 mm in diameter. The target AI was 500 for anterior segments, 400 ~ 450 for roof, and 350 ~ 400 for inferior and posterior segments. If AF maintained after CPVI, electrical cardioversion was performed to restore sinus rhythm. Additional substrate ablation was performed if there were low‐voltage areas after voltage mapping of the left atrium.^11^

### 2.4 Evaluation of procedure parameters by AIFV system

All the electrophysiologists were blinded to the research analysis and evaluation results. All the clinical characteristics, intraprocedural data, and peri‐procedural complications were all recorded and off‐line analyzed.

Ablation points on 3D mapping were recorded by Visitag system (Visitag^R^, Biosense Webster Inc.). VISITAG settings included the following: (a) catheter stability range of motion ≤2.5 mm, (b) catheter stability duration >3 s, and (c) CF ≥5 g with time ≥ 25%. An ablation–evaluation software named AIFV (using AI to analyze the raw data from CARTO3) system (Biosense Webster Inc. Irvine, CA) was used to describe the overall quality for the two pulmonary vein (PV) circles. In summary, the software gives a statistical delineation from six main parameters based on the data derived from the CARTO3 system. Six main parameters were defined as follows:

1. Break point was referred to condition that any inter‐lesion distance ≤3 mm.
2. Gap was referred to condition that any inter‐lesion distance ≥6 mm.
3. Percentage of lesions with effective force‐over‐time (FOT) ratio was defined as lesions with effective FOT (with force above 5 g for more than 40% of the time) over all lesions in PV circles.
4. Percentage of lesions with effective AI (AI ratio) was defined as lesions with effective AI (300 ~ 600) over all lesions in PV circles.
5. Total PVI time was defined as the duration from the first radiofrequency (RF) delivery of PVI to the last RF delivery of PVI.
6. RF time ratio was defined as the total RF time during PVI over the total PVI procedure time.

### 2.5 Long‐term follow‐up

All patients were administered anti‐arrhythmic agents for 3 months if there was no contraindication and were followed in the outpatient clinic after the ablation. Clinical evaluation and 24‐h Holter recordings were performed at 1, 3, 6, and 12 months after the procedure. The 12‐lead surface electrocardiogram was performed at any time if the patients reported arrhythmic symptoms. Recurrence was defined as atrial tachyarrhythmia lasting over 30 s after a 3‐month blanking period during the follow‐up.

### 2.6 Statistical analysis

Continuous data are presented as mean ± SD, and categorical data are described as counts and percentage. To compare differences between the two groups, Student's *t*‐tests and chi‐square tests were performed, as appropriate. Propensity score matching analysis was used to match each patient from the general anesthesia (GA) group with a patient from the conscious sedation (CS) group in a 1:1 fashion, with a caliper of 0.1 and no replacements. Since the six parameters have different units and orders of magnitude, in the present study, data were scaled using the function “scale” of the R package “base” and visualized with the radar plots. Negative values were used to represent the negative correlation between the number of gaps, break point rate, and total PVI time with PVI lesion quality, to enable a more intuitive visualization. Data were analyzed using R version 4.1.3.

## 3 RESULTS

### 3.1 Patient population

From June 2020 to June 2021, a total of 36 patients underwent ablation under GA were enrolled in GA group, while 109 patients who received AF ablation under CS by the same group of electrophysiologists during the same period were enrolled in CS group. The baseline characteristics of the enrolled patients are summarized in Table 1. Patients in the CS group had a higher rate of nonparoxysmal atrial fibrillation. There was no significant difference between these two groups regarding age, gender, CHA2DS2‐VASc scores, and cardiac function evaluated by echocardiogram. The prevalence of hypertension, coronary artery disease, diabetes, prior ischemic stroke, and heart failure was also comparable between the two groups. Propensity score matching was performed with the following baseline variables: age, gender, AF types, hypertension, coronary artery disease, diabetes, prior ischemic stroke, heart failure, CHA2DS2‐VASc score, and several echocardiography parameters. After propensity score matching, a total of 58 patients (29 patients in the CS group and 29 patients in the GA group) were available for further analyses of procedural parameters. Totally 6/29 patients in GA group and 5/29 patients in CS group received additional substrate ablation. The medium follow‐up time was 24 months (range 18–30 months).

**TABLE 1:** Baseline characteristics of enrolled patients.

	All patients			Propensity score matched		
	CS group	GA group	*p* value	CS group	GA group	*p* value
	(*n* = 109)	(*n* = 36)		(*n* = 29)	(*n* = 29)	
Age (year)	61.76 ± 10.15	62.08 ± 12.92	.878	62.28 ± 9.93	60.93 ± 13.43	.666
Male	82 (75.2%)	28 (77.8%)	.932	20 (69.0%)	22 (75.9%)	.769
NPAF	72 (66.1%)	16 (44.4%)	.035	15 (51.7%)	15 (51.7%)	1.000
Hypertension	57 (52.3%)	20 (55.6%)	.883	14 (48.3%)	15 (51.7%)	1.000
CAD	29 (26.6%)	9 (25.0%)	1.000	9 (31.0%)	8 (27.6%)	1.000
Diabetes	12 (11.0%)	7 (19.4%)	.310	3 (10.3%)	4 (13.8%)	1.000
Prior ischemic stroke	19 (17.4%)	12 (33.3%)	.075	9 (31.0%)	8 (27.6%)	1.000
Heart failure	17 (15.6%)	9 (25.0%)	.306	8 (27.6%)	7 (24.1%)	1.000
CHA2DS2‐VASc score	2.15 ± 1.74	2.75 ± 1.93	.082	2.62 ± 1.93	2.48 ± 1.90	.785
LAD (mm)	40.96 ± 4.16	40.47 ± 4.83	.556	41.48 ± 4.82	41.62 ± 3.97	.906
LVDd (mm)	47.93 ± 4.08	48.28 ± 5.01	.674	48.14 ± 3.77	48.28 ± 5.16	.908
LVEF (%)	62.26 ± 4.65	61.08 ± 5.88	.219	61.53 ± 5.38	61.48 ± 5.64	.968

*Note*: Values are mean ± SD or *n* (%).

Abbreviations: CAD, coronary artery disease; LAD, left atrium diameter; LVDd, left ventricular end‐diastolic dimension; LVEF, left ventricular ejection fraction; NPAF, nonparoxysmal atrial fibrillation.

### 3.2 Procedural parameters and long‐term outcomes

**FIGURE 1:**
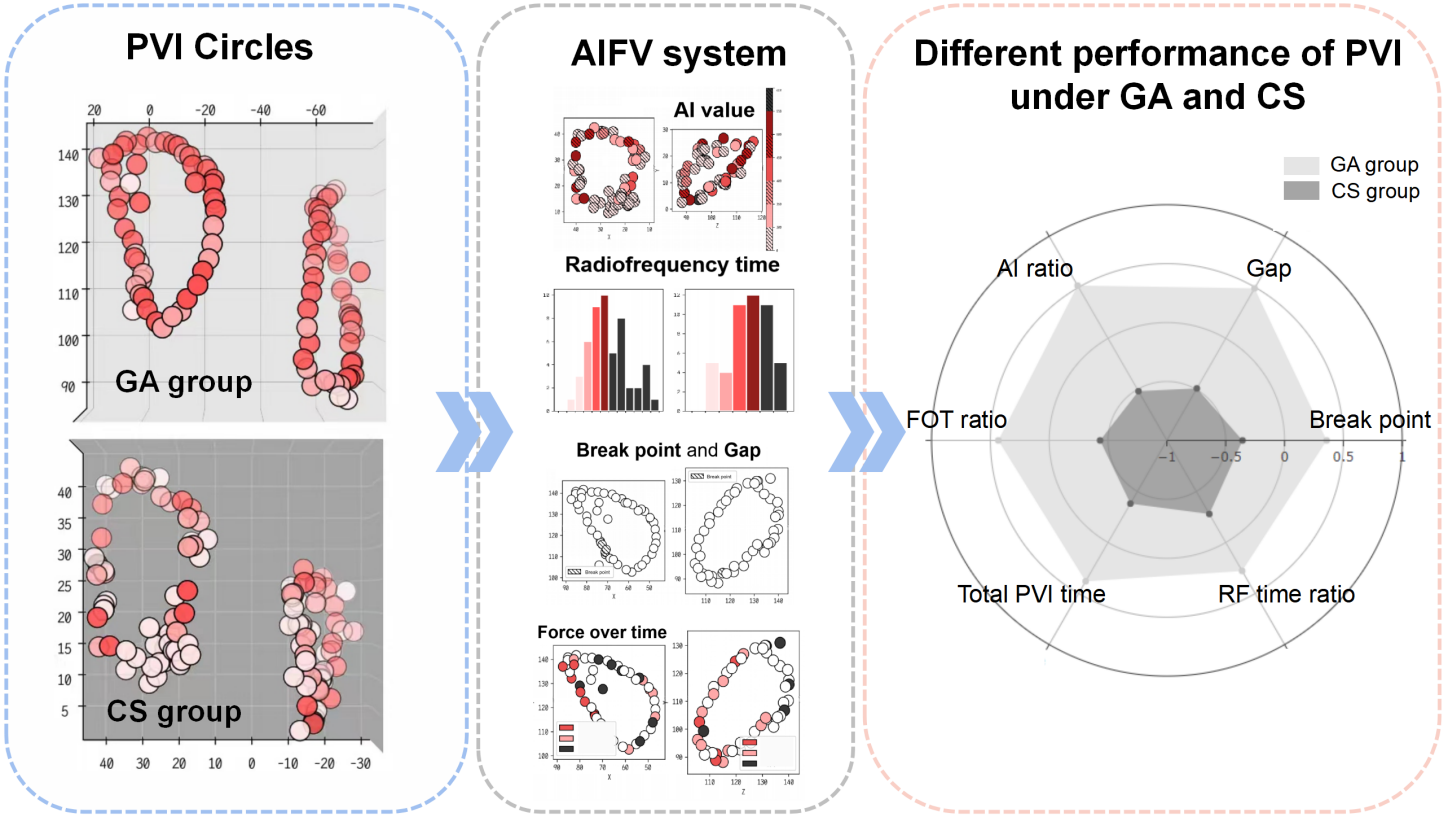
General anesthesia improves PVI performance. The AIFV system will independently evaluate each ablation point in pulmonary vein (PV) circles, including inter‐lesion distance, percentage of lesions with effective force‐over‐time (FOT ratio), percentage of lesions with effective AI (AI ratio), radiofrequency time, etc., and finally present the results in the form of a hexagonal radar chart. Ablation of atrial fibrillation under general anesthesia can comprehensively improve various procedural parameters.

Procedural characteristics are given in Table 2. PVI was obtained in all cases. Circumferential ablation had a better performance in GA group compared with that in CS group. Compared with the CS group, procedures with GA were characterized by shorter total PVI time (51.4 ± 10.6 min vs. 67.8 ± 26.4 min; *p* = .003), and higher RF time ratio (62.6 ± 11.2% vs. 55.8 ± 12.4%; *p* = .032). The number of gaps in the GA group and CS group was 1.0 ± 1.1 and 3.0 ± 2.2, respectively (*p* < .001). The GA group also showed a higher effective AI ratio (87.5 ± 6.0% vs. 74.1 ± 14.5%; *p* < .001) and effective FOT ratio (85.3 ± 8.8% vs. 69.2 ± 22.3%; *p* = .001) and a lower rate of break point (12.8 ± 9.3% vs. 22.5 ± 15.4%; *p* = .005). The difference of the parameters between these two groups was presented in radar charts (Figure 1).

**TABLE 2:** Procedural parameters of the two groups.

	CS group (*n* = 29)	GA group (*n* = 29)	*p* value
Break point rate (%)	22.45 ± 15.44	12.76 ± 9.32	.005
Gap	2.97 ± 2.21	1.00 ± 1.13	<.001
AI ratio (%)	74.14 ± 14.48	87.48 ± 6.01	<.001
FOT ratio (%)	69.16 ± 22.33	85.31 ± 8.75	.001
Total PVI time (min)	67.83 ± 26.35	51.41 ± 10.60	.003
RF time ratio (%)	55.83 ± 12.39	62.64 ± 11.19	.032
Recurrence	12 (41.4%)	6 (20.7%)	.156

*Note*: Values are mean ± SD or *n* (%).

Abbreviations: AI, ablation index; FOT, force‐over‐time; PVI, pulmonary vein isolation; RF, radiofrequency.

One patient in the CS group suffered from pericardial tamponade, which resolved after pericardial drainage. Two patients in the CS group reported a transient episode of facial palsy and resolved after 20 min which diagnosed to be a stroke by the magnetic resonance imaging.

**FIGURE 2:**
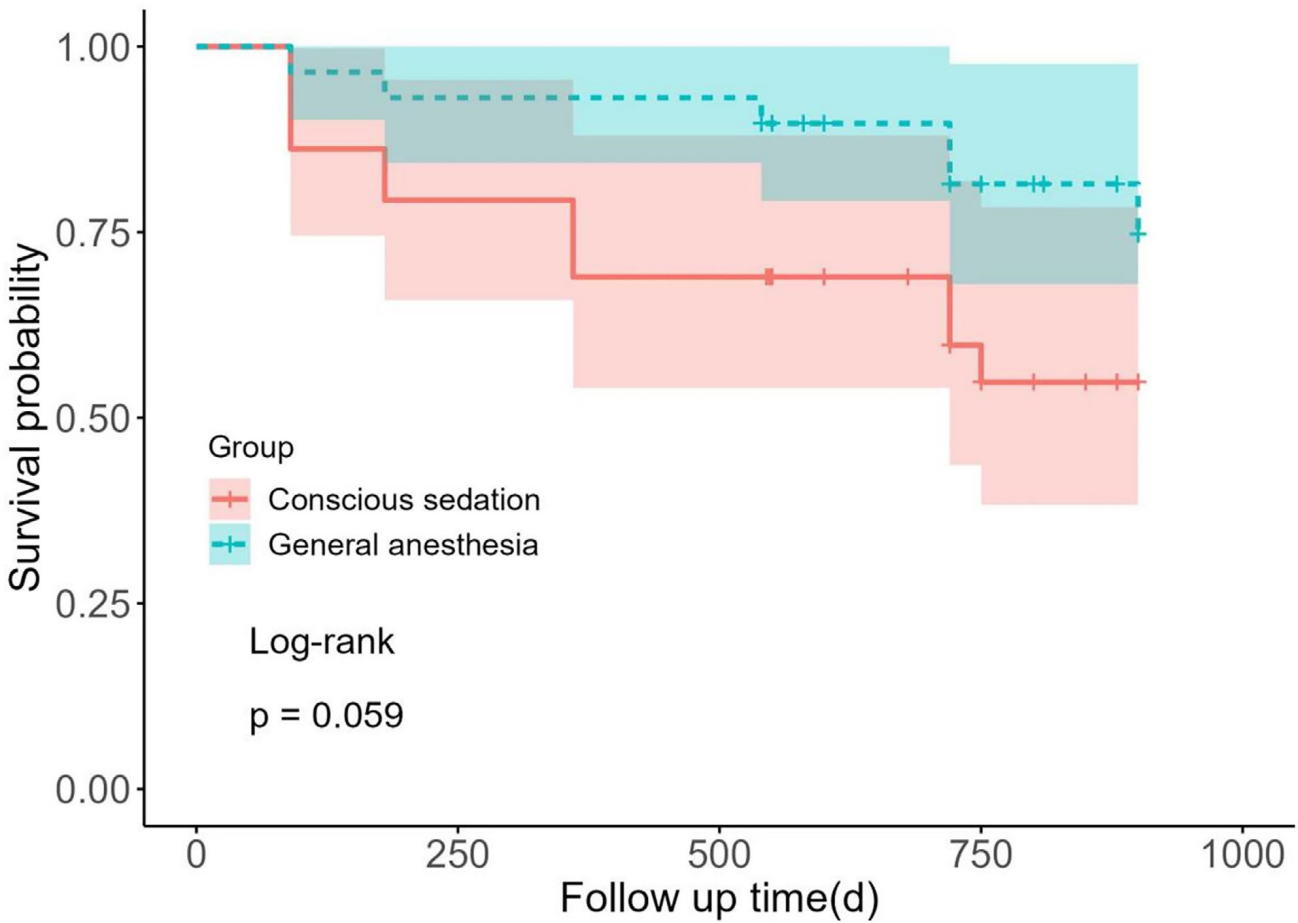
Kaplan–Meier survival curve for freedom from atrial tachyarrhythmia after a single procedure among 2 groups.

After a median follow‐up of 24 months, there was a trend toward lower recurrence rate of AF in the GA group (20.7% vs. 41.4%; *p* = .156), although the difference did not reach statistical significance (Table 2). A time‐to‐event analysis is shown in Figure 2.

## 4 DISCUSSION

This study, for the first time, comprehensively compared the circumferential pulmonary vein isolation ablation performance under GA with that under CS by a novel system named AIFV. The results indicate that GA could significantly improve the procedural efficiency and PVI quality from multiple perspectives.

### 4.1 GA improves quality of PVI

In a study by Ouyang et al., the 5‐year single‐procedure success rate of CPVI was found to be only 46.6%, while it increased to 79.5% after one (1–3) reisolation procedure.^12^ The large discrepancy in success rates between single and redo PVI highlights the pressing need to improve lesion quality during the index procedure. It has been proved that the transmurality, durability, and continuity of lesions are key indicators of PVI quality, which correlates with the long‐term AF control. Among them, the transmurality and durability depend on the performance of catheter to stick to the atrial wall under a certain contact force and delivery a certain setting of power for a certain period of time.^13^ Several indexes have been developed to evaluate the relationship between the lesion depth and volume with the contact force, ablation duration, and power setting using an experimental model.^14^, ^15^ However, in the scenario of real practice, the stick of catheter tip to the atrial wall might always shifts because of respiration, heartbeats, and even the manipulation. GA provides a regulated respiration mode with a small tidal volume, allowing for finer catheter tip movements that result in more consistent contact force and potentially more effective lesions compared to intermittent contact force.^16^ As demonstrated in our study, GA was associated with a higher effective AI ratio (>80%) and CF ratio (>70%) compared to those in the CS group. Based on our previous observations, adequate contact force and FOT ratio might be translated into more favorable control of AF,^6^ though, in the present study, the patient did not demonstrate significant difference in terms of the long‐term control of AF, which may be attributed to the small sample.

### 4.2 GA enhances procedural efficiency of PVI

During a procedure to ablate the PVs, patients may experience pain that causes movement and intermittent deep breathing. This can lead to wasted time adjusting the catheter tip position and pausing RF delivery. However, when the procedure is performed under general anesthesia (GA), patients remain still, which makes the ablation procedure smoother. Furthermore, the absence of movement related to the breath helps the manipulators to move the catheter and make the tip accessing to the target region more easily, which has been reflected by less break points, and larger RF time ratio in the GA group.

As a result, GA can shorten the duration of PVI and RF time, while increasing the RF ratio. This suggests that GA makes the PVI procedure easier to control, and less time is wasted chasing and managing gaps.

### 4.3 Potential benefit of GA beyond the efficacy and quality

Prolonged procedure duration can cause discomfort for patients and make it difficult for them to remain still. This can increase the risk of various complications. When doing the ablation on the roof of left atrial, the respiratory amplitude and movement of the patient will significantly influence the contact force. The instability of respiratory amplitude and sudden movements may instantaneously increase the contact force on the tip of the catheter, thereby significantly escalating the risks of cardiac tamponade. Additionally, using high contact force, high ablation power, and repeatedly ablating lesions in close proximity may result in excessive burns, which can potentially lead to complications such as tamponade or damage to the esophagus.^15^, ^17^ Our observations indicate that GA can provide a more favorable environment for achieving well‐placed lesions and making catheter manipulation more manageable and predictable. Better catheter stability could optimize the delivery of RF energy and avoid instantaneous changes in contact force and RF power.

All these advantages were especially important for young electrophysiologists, which might shorten the learning curve, reduce excessive damage, and decrease the incidence of manipulation‐related complications.

The benefits of general anesthesia have been substantiated, and there is a growing recognition of improved deep sedation techniques, such as total intravenous anesthesia (TIVA).^18^ Looking ahead, our future endeavors should be directed toward addressing the challenges of ensuring smooth and safe anesthesia administration in the face of a shortage of anesthesiologists, as well as optimizing operational workflows to enhance turnover efficiency.

### 4.4 Study limitations

Our study has several limitations. First, it was a nonrandomized study with a relatively small number of patients. However, patients were enrolled consecutively and propensity score matching was performed to reduce selection bias. Second, the operators and patients were not blinded to the anesthesia, which will possibly have a subtle effect on the operator's behavior during the procedure.

## 5 CONCLUSIONS

GA improves the PVI lesion quality and procedural efficiency from multiple perspectives as demonstrated by the AIFV system, which is particularly crucial for young operators.

## FUNDING INFORMATION

No funding was received for conducting this study.

## CONFLICT OF INTEREST STATEMENT

The authors have no conflict of interest to declare.

## ETHICS APPROVAL STATEMENT

The study was approved by the institutional review board.

## PATIENT CONSENT STATEMENT

Consent was obtained from all the subjects.

## PERMISSION TO REPRODUCE MATERIAL FROM OTHER SOURCES

No reproduce material from other sources.

## DECLARATIONS

**Approval of the research protocol:** The study was approved by the Institutional Ethical Review board.

**Informed Consent:** All patients provided written informed consent.

**Registry and the Registration No. of the study:** N/A.

**Animal Studies:** N/A.

## Data Availability

The data that support the findings of this study are available from the corresponding author upon reasonable request.
